# Upfront Comprehensive Genomic Profiling in Advanced Biliary Tract Cancer: Real‐World Implementation and Clinical Outcomes

**DOI:** 10.1002/cam4.72461

**Published:** 2026-11-03

**Authors:** Saskia Hussung, Mia Roth, Dilara Akhoundova, Clelia Pistoni, Judith Konrat, Franca Lisy, Tabea Ibach, Tomas Brezina, Markus Rechsteiner, Daniela Lenggenhager, Martin Zoche, Holger Moch, Achim Weber, Ralph Fritsch

**Affiliations:** ^1^ Department of Medical Oncology and Hematology University Hospital Zurich Zurich Switzerland; ^2^ University of Zurich Zurich Switzerland; ^3^ Department of Medical Oncology Inselspital, University Hospital of Bern Bern Switzerland; ^4^ Department for BioMedical Research University of Bern Bern Switzerland; ^5^ Department of Pathology and Molecular Pathology University Hospital Zurich Zurich Switzerland; ^6^ Institute of Molecular Cancer Research (IMCR) University of Zurich Zurich Switzerland

**Keywords:** biliary tract cancer, comprehensive genomic profiling, personalized treatment, precision oncology

## Abstract

**Background:**

Biliary Tract Cancers (BTCs) are difficult‐to‐treat malignancies with limited therapeutic options and dismal prognosis. Upfront comprehensive genomic profiling (CGP) is recommended for advanced BTC to identify actionable alterations, but its real‐world implementation remains underexplored.

**Methods:**

In 2019, we implemented upfront CGP for advanced BTC at our institution. Molecular profiling results were reviewed within our institutional molecular tumor board when clinically applicable. Patients were followed throughout their disease course.

**Results:**

A total of 80 patients with locally unresectable (*n* = 14) or metastatic (*n* = 66) BTC underwent CGP. Testing was performed on cytology material (6/80), tumor biopsies (38/80), or surgical specimens (36/80). In 59/80 cases (73.8%) testing was carried out prior to the beginning of palliative first‐line treatment. In 42/80 (52.5%) cases, at least one clinically actionable genomic alteration was identified. The most frequent actionable alterations involved *IDH1* (13.8%), *FGFR2* (8.8%), *ERBB2* (HER2) (5.0%), *KRAS* G12C (2.5%), and *BRAF* (1.3%). Alterations in DNA damage response genes were detected in 11/80 tumors, and 3/80 were dMMR/MSI‐H. *FGFR2* and *IDH1* mutations were detected in ICC only, while HER2 alterations were more commonly detected in ECC and GBC. 11/42 patients with a clinically actionable alteration received targeted treatment during the disease course (1 first‐line, 10 later lines). Among patients with an evaluable preceding systemic treatment line, treatment duration was longer with molecularly matched treatment than with the immediately preceding treatment line (*p* = 0.0391). 2/14 patients with initially unresectable BTC achieved secondary resectability during molecularly matched treatment.

**Conclusion:**

Upfront CGP is feasible in routine clinical practice and identifies clinically actionable alterations in a substantial proportion of patients with advanced BTC. However, only a subset received molecularly matched treatment, highlighting a gap between molecular actionability and treatment implementation. Timely access to matched therapies remains critical to the clinical utility of precision oncology in BTC.

**Abbreviations**
BRAF: B‐Raf proto‐oncogene
BRCA: breast cancer gene
BTC: biliary tract cancer
CGP: Comprehensive genomic profiling
CI: confidence interval
DDR: DNA damage response
dMMR: mismatch repair deficient
DNA: deoxyribonucleic acid
ECC: extrahepatic cholangiocarcinoma
EMA: European Medicines Agency
ESMO: European Society for Medical Oncology
FDA: US Food and Drug Administration
FFPE: formalin‐fixed, paraffin‐embedded
GBC: gallbladder cancer
HR: hazard ratio
ICC: intrahepatic cholangiocarcinoma
IRB: institutional review board
KRAS: Kirsten rat sarcoma virus
MMR: mismatch repair
MSI‐H: microsatellite instability‐high
NCCN: National Comprehensive Cancer Network
NGS: next generation sequencing
OS: overall survival
PD: progressive disease
PFS: progression‐free survival
pMMR: mismatch repair proficient
RNA: ribonucleic acid
SNV: single nucleotide variants
TMB: tumor mutational burden
UICC: Union Internationale Contre le Cancer

## 1 Introduction

Treatment of patients with biliary tract cancer (BTC) remains challenging [1]. BTCs encompass an anatomically, clinically, and molecularly heterogeneous group of tumors [2] often diagnosed at the unresectable and metastatic stage. For locally advanced tumors and metastatic disease, palliative systemic treatment remains the best‐established treatment option [3]. With the introduction of first‐line chemoimmunotherapy, median overall survival for molecularly unselected patients with advanced BTC has surpassed 12 months in Phase III trials [4, 5].

Importantly, next generation sequencing (NGS) has uncovered a range of actionable molecular driver alterations in a relevant subgroup of BTCs [6, 7]. Clinically actionable molecular alterations are most frequent in intrahepatic cholangiocarcinoma (ICC), including *FGFR2* alterations (up to 15%), *IDH1* mutations (15%–20%), and the *BRAF* V600E mutation (3%–5%) [6, 8, 9]. In extrahepatic cholangiocarcinoma (ECC) and gallbladder cancer (GBC), *ERBB2/*HER2 amplifications, overexpression, or mutations are enriched compared with ICC [10, 11, 12]. Across all BTC subtypes, additional actionable or potentially targetable alterations include *KRAS* G12C mutations (approximately 1%) [13, 14], microsatellite instability (MSI‐H, 2%–4%) [15], and rare fusion oncogenes such as *NTRK* (< 1%) [11, 16, 17, 18]. Alterations in DNA damage response (DDR) genes, including *BRCA1* and *BRCA2*, occur in a subset of BTCs and may have therapeutic and hereditary implications; however, their predictive value for PARP inhibition in BTC remains insufficiently established [19]. ECCs and GBCs have a high proportion of currently non‐targetable molecular alterations in *KRAS* (30%–45%) and *TP53* (30%–50%) [6, 9].

Biomarker‐directed therapies, including FGFR inhibition in *FGFR2* fusion‐ or rearrangement‐positive cholangiocarcinoma and combined BRAF/MEK inhibition in *BRAF* V600E‐mutated BTC, have demonstrated clinically meaningful response rates [1, 20, 21, 22, 23]. More recently, HER2‐directed therapies have also shown substantial antitumor activity in molecularly selected BTC populations [11, 18]. However, randomized evidence demonstrating a survival benefit from molecularly targeted therapy in BTC remains limited, with Phase III evidence available for ivosidenib in previously treated *IDH1*‐mutated cholangiocarcinoma [24, 25]. To identify therapeutically relevant alterations in a timely manner, current guidelines recommend comprehensive molecular profiling in patients with locally advanced unresectable or metastatic BTC when first‐line systemic treatment is initiated. Molecular profiling should therefore ideally be performed before or during first‐line therapy to ensure that potentially actionable findings are available when subsequent treatment decisions are required [3, 17, 26, 27, 28]. The increasing number of biomarker‐directed treatment options further emphasizes the importance of obtaining a comprehensive molecular profile early in the disease course.

However, detecting relevant alterations in BTCs can be technically challenging. The molecular landscape of activating genomic *FGFR2* alterations is complex, and some routine next generation sequencing (NGS) assays may not cover all relevant alterations [29, 30]. Additionally, the low tumor cell content of many BTCs can sometimes complicate analysis. Negative results, especially in ICC without other detectable oncogenic drivers, should always be questioned, and the analysis might need to be repeated, expanded, or supplemented. Although liquid biopsy may complement tissue‐based molecular profiling when tumor material is limited, the sensitivity for detecting genomic rearrangements and gene fusions may depend on circulating tumor DNA abundance and assay characteristics, warranting cautious interpretation of negative results [31]. Ambiguous results should also be reviewed by a molecular tumor board, as many *FGFR2* alterations initially deemed irrelevant are now recognized as activating and therapeutically exploitable.

At the University Hospital Zurich, comprehensive molecular tumor testing for all locally advanced, unresectable or metastatic BTCs was established in 2019. The methodology employs the FoundationOne CDx Assay, supplemented in individual cases by RNA‐based fusion investigations and immunohistochemistry assays. To evaluate the real‐world implementation of this approach, we retrospectively analyzed patients undergoing CGP between January 2019 and June 2022, focusing on the feasibility and molecular yield of CGP, subsequent molecular tumor board recommendations, implementation of molecularly matched treatment, and descriptive clinical outcomes.

## 2 Material and Methods

### 2.1 Patient Cohort

Patients with clinically and histologically confirmed BTC who underwent both tumor treatment and molecular tumor profiling at the University Hospital Zurich were included in this retrospective analysis. Patients were included during the study period from January 2019 to June 2022. Diagnosis of locally advanced or metastatic disease relied on radiological and cyto−/histological confirmation. The study comprised 80 patients, all of whom provided written informed consent for the collection and analysis of genomic data, in addition to clinic‐pathologic, treatment, and outcome data sourced from electronic medical records. Local institutional review board (IRB) approved all relevant procedures and analyses (Cantonal Ethics Committee Zurich: BASEC No. 2021–01094). This retrospective analysis was conducted in accordance with the Declaration of Helsinki and the Good Clinical Practice guidelines. Treatment was performed as per standard of care. Molecular tumor profiling results were discussed at our institution's weekly molecular tumor board when clinically applicable. Regular follow‐ups, scheduled every 2 to 3 months, encompassed both clinical assessments and radiological examinations utilizing either computed tomography or magnetic resonance imaging. Evaluations of radiological responses were determined based on the standard assessment of radiological images.

### 2.2 Molecular Tumor Profiling

Comprehensive molecular tumor profiling was performed using the FoundationOne CDx assay. FoundationOne CDx is an FDA‐approved comprehensive genomic profiling (CGP) assay that detects substitutions, insertions, deletions (indels), and copy number variations across 324 genes [32, 33]. It also specifically pinpoints 36 selected gene rearrangements [32, 33]. DNA was extracted from formalin‐fixed, paraffin‐embedded (FFPE) tumor samples and subsequently sequenced using libraries selected via Hybrid Capture on the Illumina HiSeq platform. Sequencing results were interpreted using a customized analysis pipeline. Alongside diverse genomic alterations, the assay also provides insights into genomic indicators like MSI‐H, and the tumor's mutational burden (TMB).

All NGS diagnostics were performed at the Department of Pathology and Molecular Pathology at the University Hospital Zurich in accordance with accredited diagnostic procedures.

### 2.3 Statistical and Survival Analysis

Primary endpoint of this retrospective analysis was the prevalence and frequency of clinically actionable molecular alterations. Clinical actionability was defined as the potential for targeting with FDA‐approved drugs, either in label or off‐label, associated with expected clinical benefit. Molecularly matched treatment was defined as systemic anticancer therapy selected specifically on the basis of an actionable molecular alteration or biomarker identified by CGP. Standard platinum‐based chemotherapy administered in patients with DDR alterations was not classified as molecularly matched treatment.

Secondary endpoints included the feasibility of CGP, molecular tumor board recommendations, implementation of molecularly matched treatment, and descriptive clinical outcomes following molecularly matched treatment. Progression‐free survival (PFS) and overall survival (OS) of the overall cohort and clinicopathological or molecular subgroups were assessed as exploratory endpoints. PFS and OS were assessed through routine imaging by the attending physician and radiologist. PFS was defined as time from start of palliative first‐line treatment to the verified first radiologic progression based on standard restaging imaging or death due to any cause. For within‐patient analyses, PFS2 was defined as the time from initiation of molecularly matched treatment until radiological disease progression or death from any cause. PFS1 was defined as the time from initiation of the systemic treatment line immediately preceding molecularly matched treatment until radiological disease progression or death from any cause. Patients receiving molecularly matched treatment as first‐line therapy or without an evaluable preceding systemic treatment line were excluded from the PFS2/PFS1 analysis. A PFS2/PFS1 ratio > 1.3 was considered a previously proposed benchmark for clinical benefit based on published literature [34]. OS was defined as the time from initial diagnosis of locally advanced or metastatic disease until death. Patients were censored on the last follow‐up date or latest on June 17, 2024. For paired within‐patient comparisons, treatment duration under molecularly matched treatment was compared with the immediately preceding systemic treatment line using the Wilcoxon matched‐pairs signed‐rank test. Descriptive statistical analyses were conducted for categorical and continuous variables of interest. Kaplan–Meier survival analyses were used to estimate PFS and OS in the overall cohort and for exploratory clinicopathological and molecular subgroup analyses. Univariate comparisons were performed using the log‐rank test, and hazard ratios with 95% confidence intervals were estimated using univariate Cox proportional hazards regression. Comparative survival analyses according to subsequent receipt of molecularly matched treatment were not performed because treatment exposure was determined after baseline and would therefore be susceptible to immortal‐time and treatment‐selection bias. For paired comparisons between treatments within the same patients, the Wilcoxon matched‐pairs signed‐rank test was used. All statistical analyses were conducted using GraphPad Prism Version 10.2.0 (GraphPad Software Inc., La Jolla, California, USA) and IBM SPSS Statistics software version 29.0.0.0 (IBM Corporation, New York, USA). All *p* values were two‐sided, with *p* < 0.05 considered significant.

## 3 Results

### 3.1 Patient Characteristics

Between January 2019 and June 2022, 80 patients with clinically and histologically confirmed locally advanced unresectable or metastatic biliary tract carcinoma were included in the study. Clinical and pathological baseline characteristics of the study cohort are summarized in Table S1. Most patients presented with ICC (45/80; 56.3%), followed by ECC (22/80; 27.5%) and GBC (10/80; 12.5%) (Figure S1A). Three cases were classified as overlap disease and therefore listed as a separate category. 71/80 (88.8%) patients with locally advanced or metastatic disease received palliative first‐line treatment, most frequently with Gemcitabine/Cisplatin (46/71 pts.; 64.8%), reflecting the standard of care during the study period before the routine implementation of chemoimmunotherapy based on the TOPAZ‐1 and KEYNOTE‐966 trials [4, 5]. Subsequent systemic treatment was administered in 35/71 patients (49.3%) in the second‐line setting, most frequently FOLFOX (*n* = 20), followed by FOLFIRI (*n* = 5), erdafitinib (*n* = 4), FOLFIRINOX (*n* = 3), pembrolizumab (*n* = 2), and 5‐FU monotherapy (*n* = 1). Thirteen patients (16.3%) received third‐line systemic treatment, including FOLFOX (*n* = 5), 5‐FU/nal‐irinotecan (*n* = 2), and pemigatinib, FOLFIRI, sotorasib, futibatinib, pembrolizumab, or ipilimumab/nivolumab (*n* = 1 each). Two patients received fourth‐line systemic treatment with FOLFIRI (*n* = 1) or 5‐FU/nal‐irinotecan (*n* = 1). Three patients received subsequent locoregional or surgical treatment, including SBRT (*n* = 2) and liver transplantation (*n* = 1). Information on subsequent treatment was unavailable for six patients, including three patients who were lost to follow‐up. 6/80 (7.5%) patients underwent best supportive care. Median first‐line PFS was 6.0 months (95% CI: 4.7–7.3 months), and median OS was 19.0 months (95% CI: 12.5–25.5). At the time of final analysis, 65/80 (81.3%) of the patients had died.

### 3.2 Comprehensive Genomic Profiling

At the time of molecular tumor testing, the majority of patients presented with metastatic disease (66/80 pts.; 82.5%; Table S1). Testing was performed on core needle biopsies (38/80; 47.5%), archived resection specimens (36/80, 45.0%), and fine needle aspirates (6/80; 7.5%) (Table S1). CGP yielded an interpretable molecular pathology report in all 80 patients, with no complete molecular profiling failures. The median estimated tumor cell content across all samples was 40.0% (95% CI 30.0%–50.0%). Microsatellite status (MSS) and TMB status were evaluable in 72/80 (90.0%) and 60/80 (75.0%) of the patients, respectively (Figure 1). The specific reasons for non‐availability of MSI or TMB results in the remaining cases could not be systematically determined retrospectively. Among samples with evaluable TMB, the median TMB was 3.0 mutations/Mb (range 0–54.0). In 59/80 cases (73.8%) testing was carried out prior to or at the beginning of palliative first‐line treatment. Median time from ordering the CGP to receiving the molecular pathology reports was 14.0 days (95% CI 13.0–16.0 days).

**FIGURE 1:**
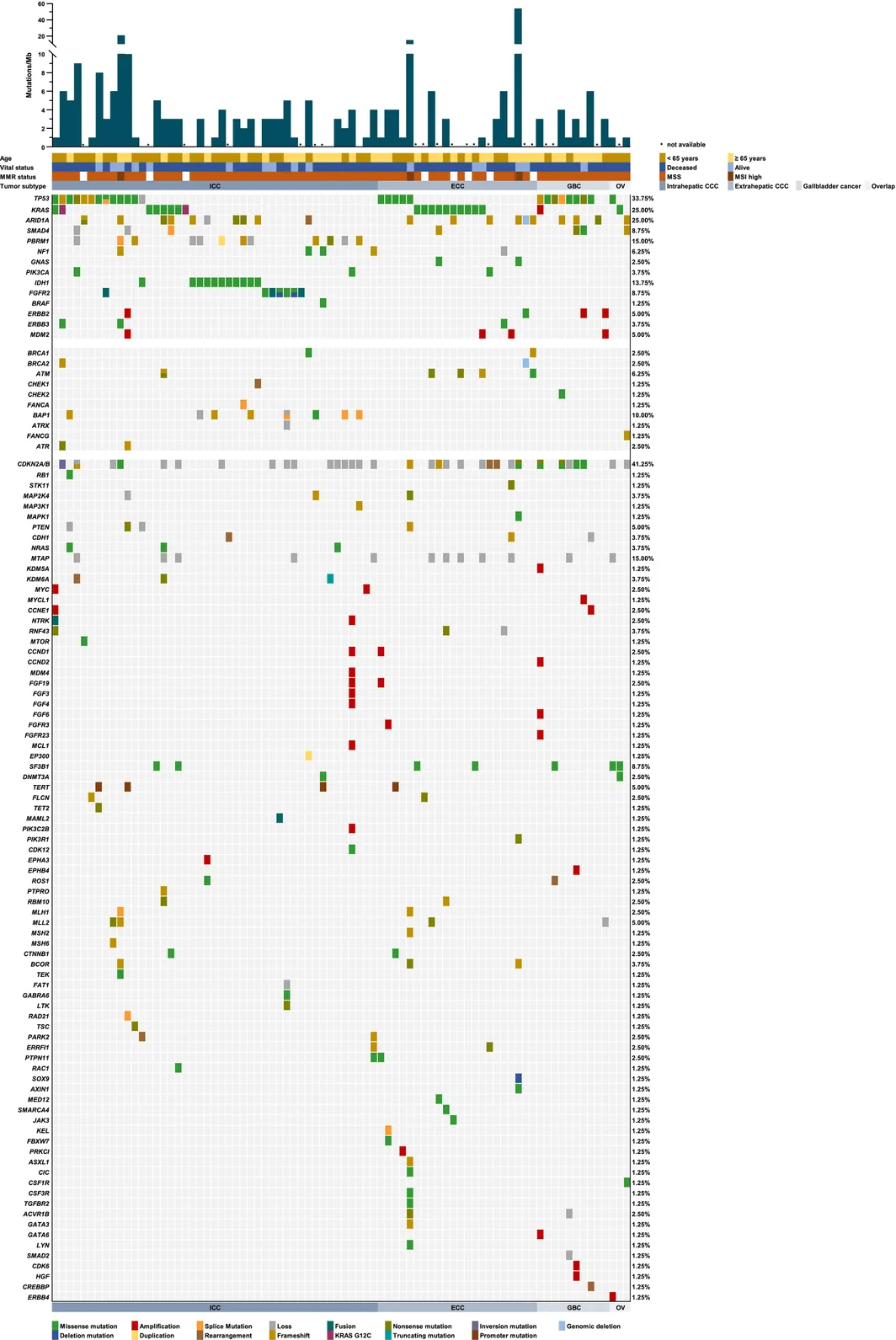
Heatmap illustrating genomic alterations detected by CGP within the BTC cohort. BTC, biliary tract cancer; CCC, cholangiocellular carcinoma; CGP, comprehensive genomic profiling; MMR, mismatch repair; MSS, microsatellite stable; MSI high, microsatellite instability.

### 3.3 Molecular Landscape and Frequency of Actionable Alterations

In our study cohort of 80 BTC patients, we identified a total of 330 molecular alterations reported as pathogenic or likely pathogenic in molecular pathology reports, with a median of 3.5 alterations (95% CI 3.0–4.0) per patient (Figure 1). The most common molecular alterations across the population were detected in *CDKN2A/B* (33/80; 41.3%), *TP53* (27/80; 33.8%), *ARID1A* (20/80; 25.0%), *KRAS* (20/80; 25.0%), two of which were *KRAS* G12C, *PBRM1* (12/80; 15.0%), *MTAP* (12/80; 15.0%), *IDH1* (11/80; 13.8%), *BAP1* (8/80; 10.0%) and *FGFR2* (7/80; 8.8%) (Figure 2A). Additionally, 11/80 (13.8%) BTCs showed alterations within DDR pathway genes, and 3/80 BTCs were dMMR/MSI‐H (Figure 2B). One patient presented with both *BRCA1* and *ATM* mutations, resulting in a total of 12 alterations within the DDR gene subset being found in the overall BTC cohort. *FGFR2* alterations (3 genomic rearrangements, 4 activating mutations, 0 high focal amps) and *IDH1* hotspot mutations were detected exclusively in ICC. In contrast, *KRAS* alterations were most frequently observed in ECC (10/22; 45.5%), while *ERBB2/*HER2 alterations were proportionally most frequent in GBC (2/10; 20.0%). Two of the three MSI‐H tumors occurred in ECC (Figure 1; Figure 2B).

**FIGURE 2:**
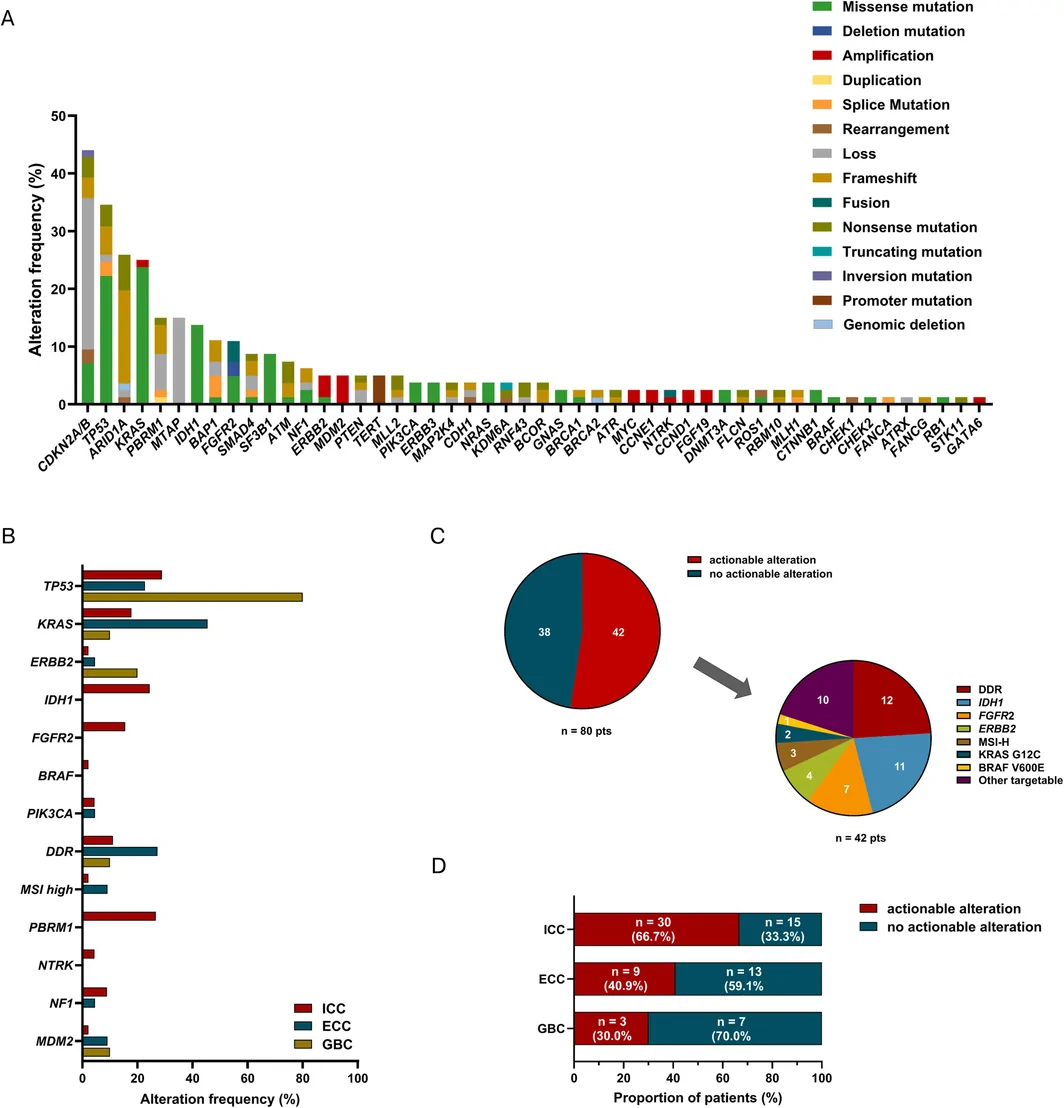
Frequency of molecular alterations across the BTC cohort. (A) The frequencies of molecular alterations within the BTC cohort are illustrated. (B) The frequencies of pathogenic molecular alterations depending on the location within the biliary tract system are illustrated. (C) Frequency and distribution of clinically actionable alterations within the BTC cohort. (D) Frequency of clinically actionable alterations depending on the location within the biliary tract system. BTC, biliary tract cancer; DDR, DNA damage response; ECC, extrahepatic cholangiocarcinoma; GBC, gallbladder cancer; ICC, intrahepatic cholangiocarcinoma.

In total, we identified 42/80 patients (52.5%) with at least one clinically actionable molecular alteration, most frequently within the DDR gene set, followed by alterations in *IDH1*, *FGFR2*, and *ERBB2/*HER2 (Figure 2C; Figure S1B). Other clinically actionable alterations included *KRAS* G12C (2/80; 2.5%) and a *BRAF* V600E mutation in 1/80 (1.3%) patients (Figure 2C).

### 3.4 Exploratory Survival Analyses by Clinicopathological and Molecular Subgroups

Overall, the majority of actionable alterations were detected in ICC (30/42; 71.4%), followed by ECC (9/42; 21.4%) and GBC (3/42; 7.1%) (Figure 2D). In exploratory survival analyses, patients with ICC had longer OS than patients with GBC (median 25 vs. 8.5 months; HR 0.35 [95% CI, 0.17–0.76; *p* = 0.0019]; Figure S1C) and showed a trend towards longer OS compared to patients with ECC (median 25 vs. 15 months; HR 0.62 [95% CI, 0.35–1.15; *p* = 0.1210]; Figure S1C). Patients with *FGFR2*‐altered tumors had longer OS than patients without *FGFR2* alterations (median 38 vs. 16 months; HR 0.36 [95% CI, 0.11–0.88; *p* = 0.0381]; Figure S1D). Patients with MSI‐H tumors showed numerically longer OS than patients with MSS tumors (median not reached vs. 18 months; Figure S1E); however, this comparison was descriptive given the small number of MSI‐H cases. There was no significant difference in OS between patients with and without *IDH1* alterations (HR 0.86 [95% CI, 0.41–1.62]; *p* = 0.6515; Figure S1F).

*Molecularly informed treatment recommendations and implementation* 68/80 (85.0%) patient cases were discussed at our institution's molecular tumor board. A personalized treatment recommendation based on CGP was given in 42/80 (52.5%) cases. Molecularly matched treatment was subsequently implemented in 11/42 patients (26.2%) harboring at least one clinically actionable molecular alteration (1/11 in the first‐line, 10/11 in later lines) (Figure 3A). In the only patient receiving molecularly matched treatment in the first‐line setting, CGP was ordered 6 days after diagnosis. First‐line systemic therapy was initiated 8 days after CGP was ordered, and the molecular pathology report became available after 18 days. Trastuzumab was subsequently added based on the molecular profiling result. Alterations in DDR pathway genes resulted in a recommendation for platinum‐based chemotherapy in 11/80 patients (13.8%), which was implemented in 8/11 patients (72.7%). As platinum‐based chemotherapy represents conventional cytotoxic treatment, these patients were not classified as having received molecularly matched treatment. PARP inhibition (in‐label or off‐label) was recommended in 8/80 patients (10.0%) with DDR alterations but was not implemented in any patient.

**FIGURE 3:**
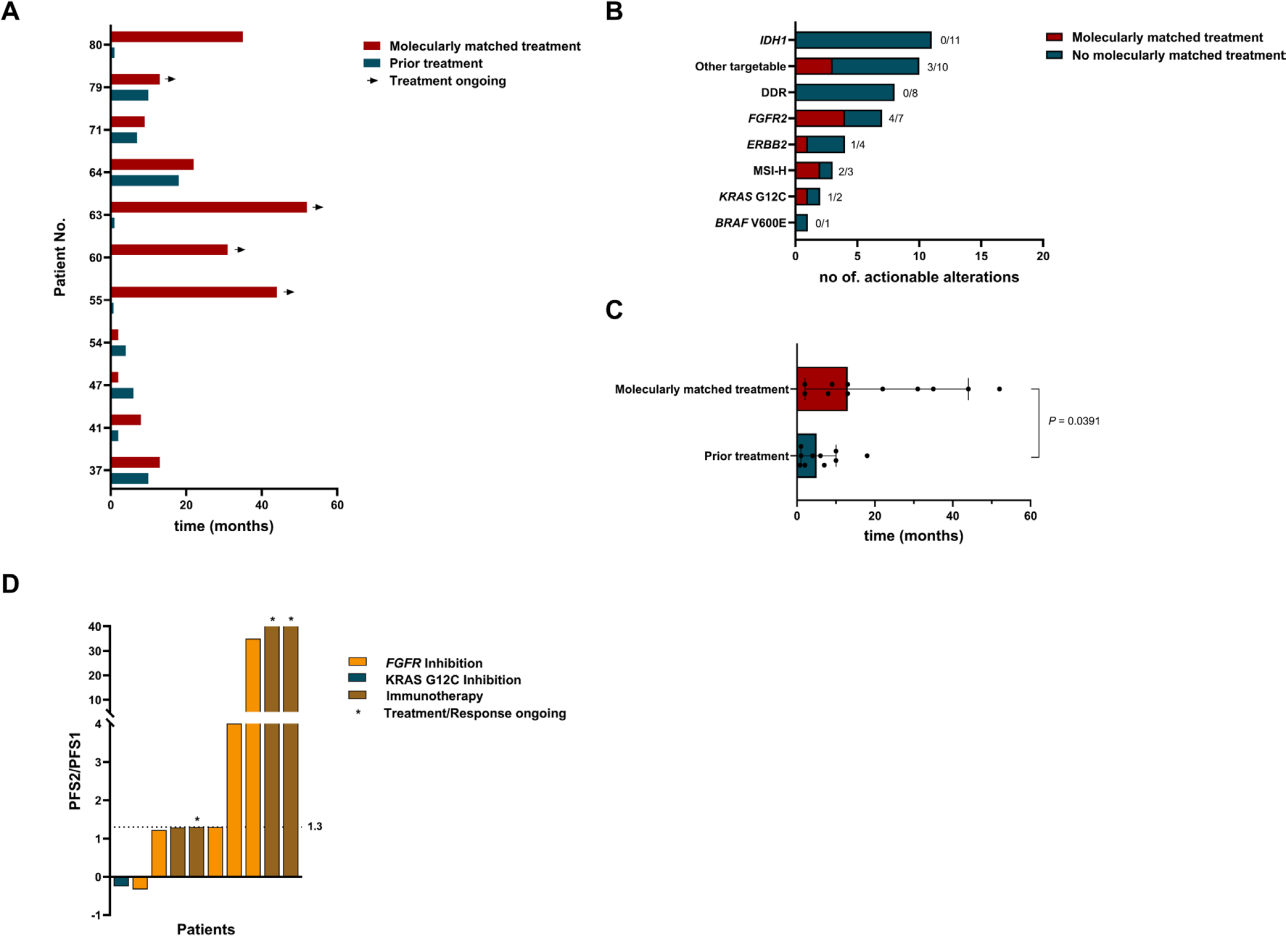
Actionable alterations and molecularly matched treatment. (A) Swimmer plot of PFS of BTC patients with actionable alterations receiving molecularly matched treatment (red) compared to the prior systemic treatment line (blue). (B) Number of actionable alterations and frequency of molecularly matched treatment. (C) PFS of BTC patients with actionable alterations receiving molecularly matched treatment (red) compared to the prior systemic treatment line (blue). (D) PFS2/PFS1 ratio of molecularly matched treatment. BTC, biliary tract cancer; DDR, DNA damage response; PFS, progression‐free survival.

Based on the results of the ClarIDHy Phase III Trial, treatment with ivosidenib was recommended in all 11/80 cases with an IDH1 hotspot mutation. However, due to the lack of Swissmedic approval at that time, none of the patients in our BTC cohort received molecularly matched treatment with an IDH1 inhibitor during the disease course (Figure 3B). FGFR2 inhibition was recommended in 7/80 (8.8%) cases and implemented in 4/7 (57.1%) patients, all as later‐line treatments. Of the 3 MSI‐high cases with a recommendation for immunotherapy, 2/3 (66.7%) received molecularly matched treatment during disease course. Additionally, immunotherapy was recommended in 2/80 (2.5%) cases with an elevated TMB and implemented in one of the patients. Sotorasib was recommended in both patients with *KRAS* G12C‐mutated tumors and implemented in one. Similarly, molecularly matched treatment with combined BRAF and MEK inhibition was recommended in 1/80 (1.3%) cases with a *BRAF* V600E‐mutated BTC. Due to subsequent treatment outside our institution, it remains unclear whether personalized treatment was implemented in the further course of disease. Additional germline testing beyond *BRCA1/2* was recommended in 2/80 (2.5%) patients based on molecular profiling results. Among the remaining 31 patients with actionable alterations, the main reasons for non‐implementation or inability to confirm implementation of molecularly matched treatment were lack of drug availability (*n* = 12), death before a subsequent treatment opportunity (*n* = 11), treatment outside our institution precluding confirmation of treatment implementation (*n* = 5), and absence of disease progression requiring further systemic therapy at data cut‐off (*n* = 2). In one patient, no specific reason was documented (Table S2).

Among patients with an evaluable systemic treatment line immediately preceding molecularly matched treatment, treatment duration under molecularly matched therapy was longer than during the preceding treatment line (median 13 vs. 5 months, *p* = 0.0391; Figure 3C). The median PFS ratio was 1.30 (Figure 3D). Among the 14 patients with initially unresectable BTC, two achieved secondary resectability during molecularly matched treatment (Figure 4A,B).

**FIGURE 4:**
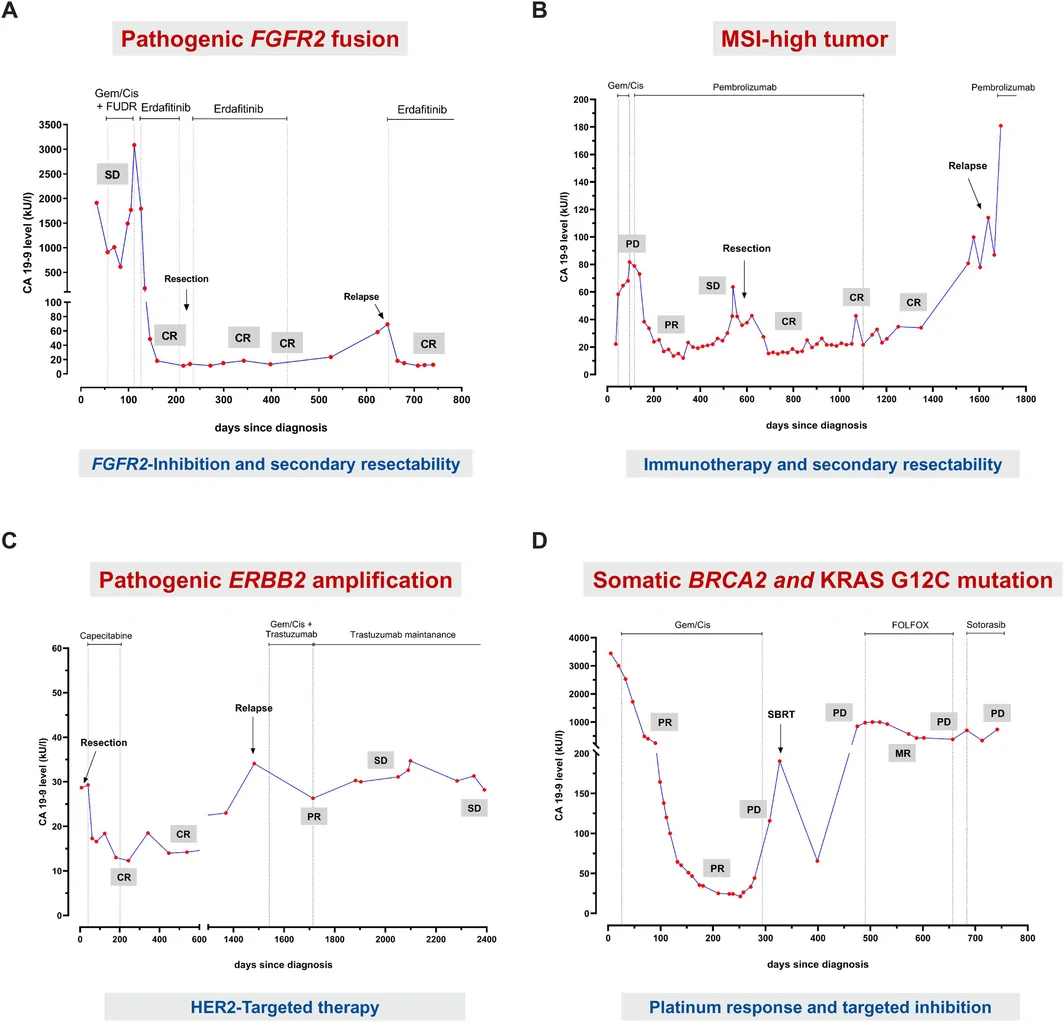
Case reports. The tumor marker course, together with radiological responses and applied systemic treatments, is depicted for four individual cases of unresectable or metastatic BTC with actionable alterations detected by CGP. (A) A 66‐year‐old patient with ICC and an *FGFR2* fusion (*FGFR2*(17) *‐ SHTN1*(7)). The patient received second‐line treatment with erdafitinib and underwent tumor resection as part of an individual concept. (B) A 73‐year‐old patient with MSI‐H BTC with a very good response to immune checkpoint‐inhibition with pembrolizumab. (C) A 67‐year‐old patient with GBC and an *ERBB2* amplification (CNV 72) in the tumor. Based on the CGP results, the patient received palliative first‐line treatment with gemcitabine/cisplatin and trastuzumab and, 2.5 years after the start of first‐line systemic treatment, is still receiving maintenance therapy with trastuzumab. (D) A 58‐year‐old patient with pathogenic *BRCA2* (VAF 15.2%) and *KRAS* G12C (VAF 22.7%) mutations. The patient received platinum‐based first‐line treatment, platinum re‐exposure and subsequent off‐label treatment with sotorasib. BTC, biliary tract cancer; CGP, comprehensive genomic profiling; CNV, copy number variation; GBC, gallbladder cancer; ICC, intrahepatic cholangiocarcinoma; MSI‐H, microsatellite instability.

### 3.5 Case Studies of Molecularly Matched Treatment

To illustrate the importance and potential of precision oncology, Figure 4 reports the treatment course of four index patients receiving molecularly matched treatment based on CGP. Figure 4A shows an exceptional response to second‐line targeted inhibition with erdafitinib in a 66‐year‐old patient with ICC and an *FGFR2* fusion. As part of an individual concept, the patient underwent tumor resection followed by additive treatment with erdafitinib for 6 months. One and a half years after resection, the patient finally presented with tumor relapse and bilateral pulmonary metastases, and treatment with the FGFR2 inhibitor was restarted, again resulting in complete remission. Figure 4B illustrates the response to immune checkpoint inhibition with pembrolizumab in a 73‐year‐old patient with MSI‐H BTC, resulting in secondary resectability. Figure 4C reports the disease course of a 67‐year‐old patient with GBC and an *ERBB2* amplification in the tumor. Based on the CGP results, the patient received palliative first‐line treatment with gemcitabine/cisplatin and trastuzumab and, 2.5 years after the start of first‐line systemic treatment, is still receiving maintenance therapy with trastuzumab. Figure 4D illustrates platinum‐based first‐line treatment, platinum re‐exposure and subsequent off‐label treatment with sotorasib in a 58‐year‐old patient with pathogenic *BRCA2* and *KRAS* G12C mutations.

## 4 Discussion

The relevant proportion of clinically actionable molecular alterations in BTCs and the growing number of targeted agents support early molecular tumor profiling in BTC patients undergoing palliative systemic treatment [1, 17, 35]. However, the optimal testing strategy has yet to be defined. Detecting relevant alterations in BTCs can be technically challenging as the molecular landscape of activating genomic FGFR2 alterations is complex, and some routine NGS assays may not cover all relevant alterations [29, 30, 36]. Additionally, the low tumor cell content of many BTCs can sometimes complicate analysis. Our data show that CGP of locally advanced, unresectable, or metastatic BTCs is feasible in a timely manner in clinical practice and uncovers potentially actionable alterations in a relevant subset of patients. Similar results employing CGP in BTCs were recently reported from a retrospective German cohort [37]. Our data confirm these findings, and with a turnaround time of 14 days from ordering the CGP to receiving the molecular pathology reports, we provide proof of clinical feasibility of such an approach. Importantly, our study extends previous real‐world observations by evaluating not only the feasibility and molecular yield of CGP, but also the subsequent clinical pathway from molecular tumor board recommendations to implementation of molecularly matched treatment and patient outcomes. This approach allowed us to demonstrate a relevant gap between the identification of actionable alterations and their actual translation into personalized treatment in routine clinical practice. Nevertheless, molecularly matched therapies could be implemented in a subset of patients, and selected patients experienced clinically meaningful treatment courses, including secondary resectability in two initially unresectable cases. Thus, our findings provide real‐world evidence that the clinical value of upfront CGP extends beyond the identification of actionable alterations and is closely linked to the timely implementation and availability of matched therapies. The molecular landscape in our cohort was consistent with the current literature [6, 9, 36, 38]. Current guidelines from major oncology organizations, including NCCN and ESMO, recommend molecular testing for locally advanced or metastatic BTCs before or during first‐line systemic treatment to ensure that actionable results are available for subsequent treatment decisions [3, 17, 28].

However, despite the promising potential of CGP, several challenges and limitations must be addressed to optimize its clinical utility. One major concern is the accessibility and affordability of CGP. While the cost of NGS has decreased significantly over the years, it remains a considerable expense for many healthcare systems, particularly in low‐ and middle‐income countries. Additionally, the integration of CGP into clinical practice requires robust bioinformatics support and multidisciplinary collaboration. Interpreting the vast amount of data generated by CGP demands sophisticated computational tools and expertise in molecular pathology, oncology, and genomics. Another critical issue is tumor heterogeneity, which may require multiple sampling sites to obtain a comprehensive molecular profile. Negative results, especially in ICC, where a high proportion of clinically actionable alterations can be found, should always be questioned. The analysis might need to be repeated, expanded, or supplemented. Moreover, resistance mechanisms during targeted therapy may develop over time, necessitating repeated biopsies and CGP to monitor changes in the molecular landscape and adapt treatment strategies accordingly. Molecular tumor boards play a crucial role in translating CGP findings into treatment recommendations, helping to navigate the complexities of molecular data, identify the most relevant alterations, and recommend appropriate targeted therapies or clinical trials. In our study, 68/80 (85.0%) patient cases were discussed at our institution's molecular tumor board, underlining the successful implementation of CGP in clinical practice. Overall, more than 50% of all BTC patients received a personalized treatment recommendation based on CGP, emphasizing the importance of molecular profiling in those patients. Alterations in DDR pathway genes frequently resulted in recommendations for platinum‐based chemotherapy or PARP inhibition. Importantly, although platinum‐based chemotherapy was considered a molecularly informed treatment option in patients with DDR alterations, it represents conventional cytotoxic therapy and was therefore not classified as molecularly matched treatment in the revised analysis. PARP inhibition was recommended in selected patients with DDR alterations but was not implemented in our cohort. However, the predictive relevance of DDR alterations for PARP inhibitor sensitivity in BTC remains insufficiently established, and prospective studies are needed to define the clinical benefit of PARP‐based treatment strategies in molecularly selected patients [39]. Furthermore, alterations in other DDR genes, including *PALB2* and *ATM*, may represent potential therapeutic vulnerabilities, although their predictive relevance for PARP inhibition in BTC remains insufficiently established [3]. In selected patients with *BRCA1/2*‐ or *PALB2*‐altered BTC who have demonstrated a favorable response to platinum‐based chemotherapy, off‐label PARP inhibition may be considered on an individual basis [3]. Beyond therapeutic relevance, the identification of DDR or Lynch‐associated alterations may also warrant genetic counseling and consideration of confirmatory germline testing in selected patients.

Molecularly matched treatment was implemented in 11/42 patients (26.2%) harboring a clinically actionable molecular alteration. The fact that only approximately one quarter of patients with actionable alterations ultimately received a matched therapy illustrates the persisting gap between molecular actionability and treatment implementation in routine clinical practice. Several factors may contribute to this gap, including treatment availability, regulatory approval, clinical condition, treatment line, and access to clinical trials. For example, although ivosidenib was recommended for patients with *IDH1*‐mutated tumors, it was not available for routine use in Switzerland during the study period because of the regulatory status at that time. Expanding access to matched therapies may therefore increase the proportion of patients in whom actionable molecular findings can be translated into treatment.

Our study has several limitations. These include the relatively small cohort size, single‐center design, and retrospective nature of the analysis. Treatment allocation was non‐randomized and occurred at different time points during the disease course, making comparisons according to subsequent receipt of molecularly matched treatment susceptible to treatment‐selection and immortal‐time bias. In addition, relevant prognostic factors could not be comprehensively adjusted for given the limited cohort size. We therefore refrain from drawing causal conclusions regarding a survival benefit of molecularly matched treatment and interpret the clinical outcome data as descriptive and hypothesis‐generating. Within‐patient comparisons of treatment duration and PFS provide exploratory information on the clinical activity of molecularly matched therapy but require confirmation in prospective studies.

## 5 Conclusion

Our real‐world data demonstrate that upfront CGP is feasible in patients with advanced BTC and can be integrated into routine clinical practice through multidisciplinary molecular tumor board evaluation and subsequent treatment recommendations. Clinically actionable alterations were identified in a substantial proportion of patients; however, only a subset ultimately received molecularly matched treatment, highlighting a relevant gap between molecular actionability and treatment implementation. Timely access to matched therapies remains an important determinant of the clinical utility of precision oncology in BTC. Prospective studies are needed to determine the impact of molecularly guided treatment strategies on clinical outcomes and to further define the optimal testing strategy.

## Author Contributions

**Saskia Hussung:** conceptualization, investigation, writing – original draft, writing – review and editing, visualization, validation, methodology, formal analysis, data curation, resources. **Mia Roth:** data curation, resources, writing – original draft, writing – review and editing. **Dilara Akhoundova:** conceptualization, writing – original draft, writing – review and editing. **Clelia Pistoni:** data curation, resources, writing – review and editing. **Judith Konrat:** writing – review and editing. **Franca Lisy:** writing – review and editing. **Tabea Ibach:** writing – review and editing. **Tomas Brezina:** writing – review and editing, resources, data curation. **Markus Rechsteiner:** writing – review and editing, software, validation. **Daniela Lenggenhager:** software, validation, writing – review and editing. **Martin Zoche:** validation, software, writing – review and editing. **Holger Moch:** validation, writing – review and editing, software. **Achim Weber:** software, validation, writing – review and editing. **Ralph Fritsch:** conceptualization, methodology, funding acquisition, supervision, writing – review and editing, writing – original draft, project administration, resources.

## Funding

The authors have nothing to report.

## Ethics Statement

The study was conducted in accordance with the principles of Good Clinical Practice and the Declaration of Helsinki. Ethical approval for all relevant study procedures and analyses was granted by the Cantonal Ethics Committee Zurich (BASEC No. 2021–01094). Written informed consent was obtained from all participants for the collection and analysis of genomic data and for the use of clinicopathological, treatment, and outcome data.

## Consent

All patients provided written informed consent to the collection and analysis of genomic and clinical data.

## Conflicts of Interest

The authors declare no conflicts of interest.

## Supporting information


**Figure S1:** Kaplan–Meier survival analyses.
(A) Frequency of different BTC subtypes. (B) Frequency of DDR alterations within the BTC cohort. (C) OS is shown for three patient subgroups: patients with ICC, ECC and GBC. (D) OS depending on *FGFR2* alteration status. (E) OS depending on MMR status. (F) OS according to *IDH1* alteration status. BTC, biliary tract cancer; DDR, DNA damage response; ECC, extrahepatic cholangiocarcinoma; GBC, gallbladder cancer; ICC, intrahepatic cholangiocarcinoma; MMR, mismatch repair; OS, overall survival; PFS, progression‐free survival.

**Table S1:** Clinico‐pathological parameters and basic clinical characteristics of the study cohort (*n* = 80).
BSC, best supportive care; CI, confidence interval; Cis, cisplatin; RCT, radiochemotherapy.
**Table S2:** Reasons for non‐implementation or unconfirmed implementation of molecularly matched treatment in patients with clinically actionable alterations.

## Data Availability

The data supporting the findings of this study are available from the corresponding author upon reasonable request. Restrictions apply to the availability of these data due to patient privacy and institutional regulations.
