# Supplementary material for: Upfront Comprehensive Genomic Profiling in Advanced Biliary Tract Cancer: Real‐World Implementation and Clinical Outcomes
Source: Cancer Med. 2026 Nov 3;15(11):e72461. doi: 10.1002/cam4.72461 (PMC13732104; doi:10.1002/cam4.72461)
Supplement: Supplementary file 1 [file CAM4-15-e72461-s001.pdf]

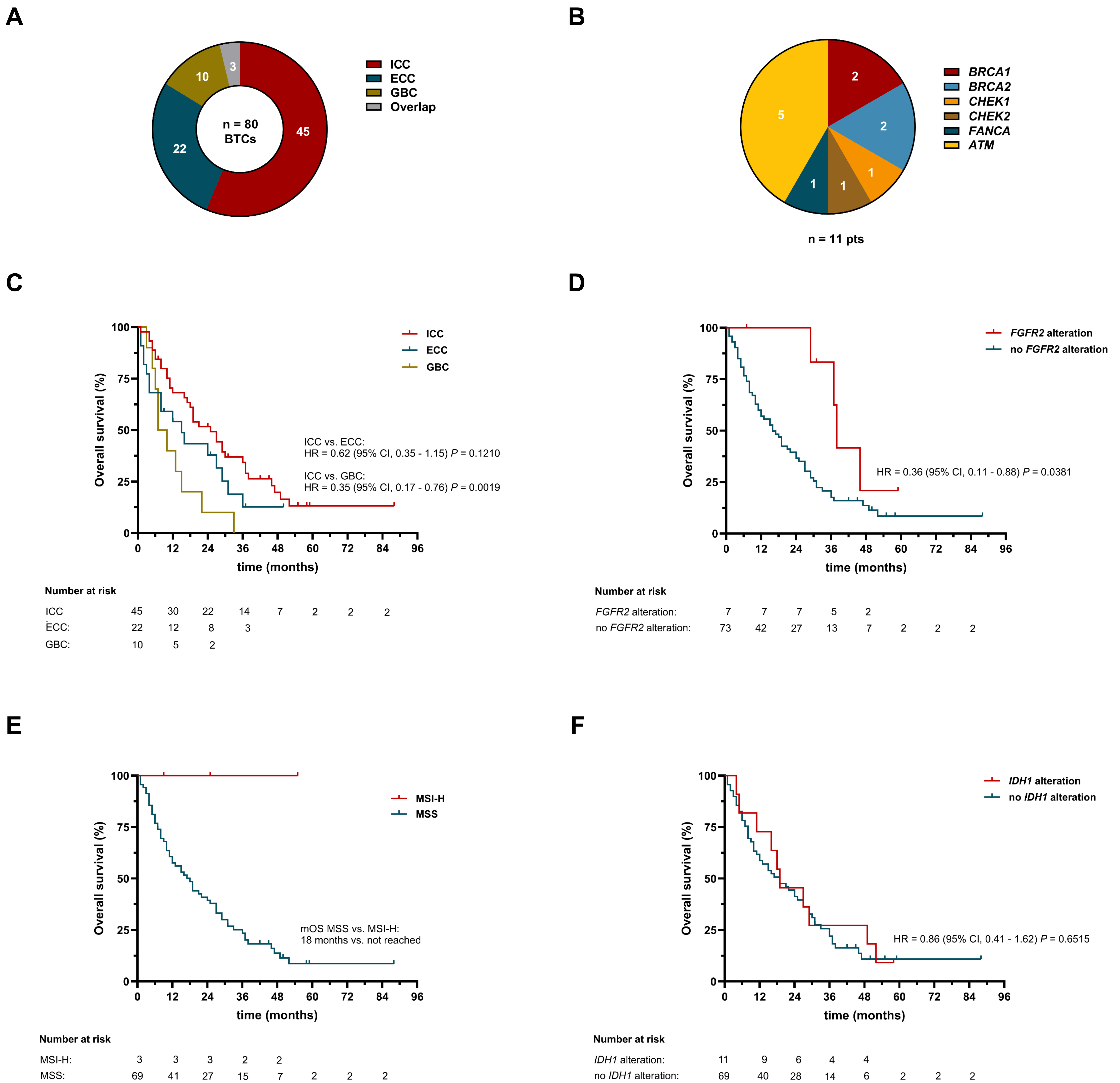
A
n = 80
BTCs
45
22
10
3
ICC
ECC
GBC
Overlap
B
2
2
1
1
1
5
BRCA1
BRCA2
CHEK1
CHEK2
FANCA
ATM
n = 11 pts
C
Overall survival (%)
time (months)
ICC
ECC
GBC
ICC vs. ECC:
HR = 0.62 (95% CI, 0.35 - 1.15) P = 0.1210
ICC vs. GBC:
HR = 0.35 (95% CI, 0.17 - 0.76) P = 0.0019
Number at risk
ICC 45 30 22 14 7 2 2 2
ECC: 22 12 8 3
GBC: 10 5 2
D
Overall survival (%)
time (months)
FGFR2 alteration
no FGFR2 alteration
HR = 0.36 (95% CI, 0.11 - 0.88) P = 0.0381
Number at risk
FGFR2 alteration: 7 7 7 5 2
no FGFR2 alteration: 73 42 27 13 7 2 2 2
E
Overall survival (%)
time (months)
MSI-H
MSS
mOS MSS vs. MSI-H:
18 months vs. not reached
Number at risk
MSI-H: 3 3 3 2 2
MSS: 69 41 27 15 7 2 2 2
F
Overall survival (%)
time (months)
IDH1 alteration
no IDH1 alteration
HR = 0.86 (95% CI, 0.41 - 1.62) P = 0.6515
Number at risk
IDH1 alteration: 11 9 6 4 4
no IDH1 alteration: 69 40 28 14 6 2 2 2
