# Supplementary material for: Upfront Comprehensive Genomic Profiling in Advanced Biliary Tract Cancer: Real‐World Implementation and Clinical Outcomes
Source: Cancer Med. 2026 Nov 3;15(11):e72461. doi: 10.1002/cam4.72461 (PMC13732104; doi:10.1002/cam4.72461)
Supplement: Supplementary file 2 [file CAM4-15-e72461-s002.docx]

**Supplemental Table 1. Clinico-pathological parameters and basic clinical characteristics of the study cohort (n = 80).**

|  | Number of patients | Percentage (%) |
| --- | --- | --- |
| Gender  Male  Female | 47  33 | 58.75  41.25 |
| Pathology  Adenocarcinoma  Carcinoma, NOS | 70  10 | 87.50  12.50 |
| Location of primary  Intrahepatic  Extrahepatic  Gallbladder  Overlap | 45  22  10  3 | 56.25  27.50  12.50  3.75 |
| Stage at molecular testing  Locally advanced  Metastatic | 14  66 | 17.50  82.50 |
| Material for molecular testing  Resection specimen  Fine needle aspiration  Core needle biopsy | 36  6  38 | 45.00  7.50  47.50 |
| First-line Treatment  Gem/Cis  Gem/Cis + Floxuridin  Gem/Cis + Durvalumab  Gemcitabine mono  FOLFOX  RCT with Capecitabine  Other  BSC  Not available (n.a.) | 46  5  3  2  3  3  9  6  3 | 57.50  6.25  3.75  2.50  3.75  3.75  11.25  7.50  3.75 |
|  | **Median** | **Range (95% CI)** |
| Age (years) | 63.5 | 25 - 84 (60 - 68) |

CI, Confidence interval

**Supplemental Table 2. Reasons for non-implementation or unconfirmed implementation of molecularly matched treatment in patients with clinically actionable alterations.**

| **Reason / implementation status** | **n** | **% of 31 patients** |
| --- | --- | --- |
| **Lack of access to recommended targeted therapy**   - IDH1 inhibitor unavailable during the study period - PARP inhibitor unavailable | 12  11  1 | 38.7  35.5  3.2 |
| **Death before a subsequent treatment opportunity**   - Death before second-line treatment - Death after second-line treatment but before planned molecularly matched therapy | 11  8  3 | 35.5  25.8  9.7 |
| **Subsequent treatment outside our institution; implementation could not be confirmed** | 5 | 16.1 |
| **No disease progression requiring further systemic therapy at data cut-off** | 2 | 6.5 |
| **No documented reason** | 1 | 3.2 |
| **Total** | **31** | **100** |

Percentages were calculated using the 31 patients with clinically actionable alterations in whom molecularly matched treatment was not implemented or could not be confirmed as the denominator.
